# Panoramic radiographic prevalence of dental developmental anomalies and jaw lesions in Mallorca, Spain: a retrospective cross-sectional study

**DOI:** 10.3389/froh.2026.1836871

**Published:** 2026-05-20

**Authors:** Daniela Vallejos-Rojas, Sandra Shabaka, Paula Llabrés, Estefanía Sparice, Pere Riutord-Sbert, Mónica Piña

**Affiliations:** 1 Faculty of Dentistry, University ADEMA School, Palma, Spain; 2 ADEMA – Health Group of University Institute for Research in Health Sciences (IUNICS), Palma, Spain

**Keywords:** apical periodontitis, dental developmental anomalies, epidemiology, jawbone lesions, panoramic radiography, tooth impaction

## Abstract

**Introduction:**

Dental developmental anomalies and jawbone lesions are frequently detected as incidental findings on panoramic radiographs. Epidemiological assessment of these conditions is clinically relevant because they may influence diagnosis, treatment planning, and preventive strategies. This study aimed to determine the radiographic prevalence and characteristics of dental developmental anomalies and jawbone lesions in patients attending a university dental clinic.

**Methods:**

A retrospective cross-sectional study was conducted using panoramic radiographs from patients aged 6–65 years who attended the ADEMA University Dental Clinic (Palma, Spain) between 2018 and 2022. Of 2,324 clinical records reviewed, 527 radiographs met the inclusion criteria. Dental developmental anomalies were classified as anomalies of number, shape, or eruption disturbances. Jawbone lesions were analyzed according to radiographic characteristics, including radiodensity and anatomical location. Associations with age and sex were also explored.

**Results:**

At least one dental developmental anomaly was detected in 38.2% of patients (201/527; 95% CI: 34.1–42.4). Eruption disturbances were the most frequent anomaly category, affecting 32.3% of patients (170/527; 95% CI: 28.4–36.4). Dental impactions were observed in 26.9% of patients (142/527; 95% CI: 23.3–30.9) and retentions in 12.5% (66/527; 95% CI: 10.0–15.6), with some individuals presenting more than one eruption disturbance. Jawbone lesions were identified in 30.0% of patients (158/527; 95% CI: 26.2–34.0), most of which corresponded to inflammatory periapical lesions (79.1%). Dental anomalies were significantly more prevalent in younger individuals, whereas jawbone lesions were more frequently observed in older patients, with the highest prevalence in the 50–65-year age group.

**Conclusion:**

A substantial proportion of patients attending this university dental clinic presented radiographically detectable dental developmental anomalies and jawbone lesions. Systematic evaluation of panoramic radiographs may therefore play an important role in early detection and clinical management of these conditions across different age groups.

## 1 Introduction

Panoramic radiography is one of the most widely used diagnostic tools in dentistry. In addition to treatment planning, it enables the detection of incidental asymptomatic findings, including developmental dental anomalies and jawbone lesions (1).

Dental developmental anomalies arise from disturbances during odontogenesis and may involve alterations in tooth number, morphology, or eruption. These findings are clinically relevant because they can complicate orthodontic and restorative planning and may be associated with functional and esthetic problems (2). Common anomalies include agenesis and supernumerary teeth affecting number, conoid teeth, dilaceration, taurodontism, fusion or gemination, and dens invaginatus affecting morphology, as well as eruption disturbances such as impaction, retention, and ankylosis (3–15).

Beyond developmental alterations, jawbone lesions constitute a heterogeneous group ranging from inflammatory periapical pathology to cystic, fibro-osseous, and neoplastic entities. Many remain clinically silent until advanced stages, making early radiographic detection essential to prevent complications such as bone destruction, tooth displacement, and morbidity (16, 17).

Taken together, developmental anomalies and jaw lesions represent frequent incidental findings in routine radiographic examinations, highlighting the importance of careful image interpretation.

From an epidemiological perspective, panoramic studies have reported highly variable prevalence rates for anomalies and lesions, reflecting differences in populations, diagnostic criteria, and imaging thresholds (18–20). European cohorts further indicate that incidental findings on panoramic radiographs are common and clinically relevant in adult populations (21).

However, most previous studies have evaluated dental anomalies and jawbone lesions separately, and there is limited evidence assessing both conditions simultaneously within the same patient cohort. This gap restricts a more integrated understanding of their distribution and potential clinical implications.

University dental clinics represent a suitable setting for this type of epidemiological assessment, as they provide access to large and diverse patient populations undergoing routine radiographic examination.

Therefore, the aim of this study was to determine the radiographic prevalence and features of dental developmental anomalies and jawbone lesions in patients treated at the ADEMA University Dental Clinic between 2018 and 2022, and to explore their association with demographic variables.

## 2 Materials and methods

### 2.1 Study design and setting

A retrospective, descriptive, single-center cross-sectional study was conducted at the ADEMA University Dental Clinic (Palma, Spain), analyzing panoramic radiographs acquired between January 2018 and December 2022 as part of routine diagnostic assessment.

A total of 2,324 clinical records from patients aged 6 to 65 years were initially reviewed. After applying eligibility criteria, 527 panoramic radiographs were included in the final sample.

All panoramic radiographs were acquired using the same digital panoramic unit (Hyperion X5, MyRay, Italy) following the standardized imaging protocol of the clinic and panoramic interpretation principles described in standard oral radiology textbooks (1).

Consecutive sampling was used, including all panoramic radiographs that met the eligibility criteria during the study period.

### 2.2 Anonymization and data management

All radiographs and clinical data were anonymized prior to analysis. Patient identifiers were removed and replaced by numerical study codes to ensure confidentiality. Only age and sex were retained as demographic variables.

### 2.3 Inclusion and exclusion criteria

Radiographs were included when they:

- belonged to patients aged 6–65 years
- were obtained for routine diagnostic purposes
- presented adequate image quality for interpretation
- had complete demographic data available

Radiographs were excluded if patients presented:

- syndromic conditions affecting craniofacial or dental development
- previous maxillofacial surgery or trauma
- incomplete clinical records
- significant radiographic distortion or artifacts

### 2.4 Examiner calibration and diagnostic consensus

Prior to data collection, examiner calibration was performed using 21 panoramic radiographs not included in the final study sample. Three examiners independently evaluated the images according to the predefined diagnostic criteria. Inter-examiner agreement with the reference examiner was substantial (Cohen's *κ* = 0.79). Discrepant cases were subsequently reviewed and discussed until consensus was reached before the start of the main evaluation. All radiographs included in the study were then assessed by the calibrated examiners under the supervision of the reference examiner to ensure consistency in interpretation.

### 2.5 Radiographic diagnostic criteria

Dental anomalies were classified into three categories: number, shape, and eruption disturbances—according to previously described radiographic criteria (3–15). Because a patient could present more than one anomaly type, these categories were not mutually exclusive.

Anomalies of number included agenesis and supernumerary teeth. Shape anomalies comprised conoid teeth, dilaceration, taurodontism, fusion or gemination, and dens invaginatus. Eruption anomalies included impaction, retention, and ankylosis.

Jawbone lesions were assessed using standardized radiographic variables including location (maxilla/mandible), size (maximum diameter in mm), shape (oval, round, irregular, pyriform), borders (diffuse, well-defined, corticated), radiodensity (radiolucent, radiopaque, mixed), and relationship to adjacent teeth (periapical, pericoronal, interradicular, or unrelated) (22, 23).

Inflammatory periapical lesions were operationally identified in this study as radiolucencies of odontogenic origin associated with deep caries, radiographic findings suggestive of pulpal pathology, or previous endodontic treatment, consistent with apical periodontitis. This category included lesions radiographically compatible with periapical granulomas and radicular cysts. Radiographically, radicular cysts typically appear as well-defined, round or oval unilocular radiolucencies, often with corticated margins in long-standing cases, whereas granulomas tend to present as smaller radiolucent lesions, usually well-defined but without corticated borders (24–26). However, this diagnosis was considered presumptive, since histopathology is required for definitive classification (27, 28).

Other jaw findings were recorded when radiographic features were suggestive of specific entities. Condensing osteitis was identified as a localized periapical radiopacity associated with teeth showing signs of chronic inflammation. Odontomas were defined as well-circumscribed radiopaque or mixed lesions with odontogenic morphology, typically surrounded by a radiolucent halo. Cemento-osseous dysplasia was recorded when lesions exhibited a characteristic mixed radiolucent–radiopaque appearance, usually in the mandibular region.

All diagnoses were based on panoramic radiographic findings and were therefore considered presumptive (22, 23, 29, 30).

### 2.6 Sample size justification

Sample size was determined to ensure adequate precision for prevalence estimation, following methodological recommendations for cross-sectional prevalence studies (31). Assuming an expected prevalence of 50%, a 95% confidence level, and a margin of error of 5%, the minimum required sample size was 387 radiographs. The final sample of 527 radiographs exceeded this requirement.

### 2.7 Statistical analysis

Continuous variables were expressed as mean ± standard deviation (SD) or median [interquartile range (IQR)], as appropriate. Normality was assessed using the Kolmogorov–Smirnov test. Comparisons were performed using Student's t-test or Mann–Whitney U test. Associations between categorical variables were evaluated using the chi-square test or Fisher's exact test.

Prevalence estimates were reported with 95% confidence intervals calculated using the Wilson method. Prevalence estimates were calculated at the patient level. Analyses describing the distribution of anomalies by tooth were performed at the tooth level and are reported descriptively. For anomaly subtypes with very low frequencies, only descriptive statistics were reported, and inferential testing was not performed due to limited statistical power.

Analyses were conducted using IBM SPSS Statistics version 20.0 (IBM Corp., Armonk, NY, USA). A two-tailed *p*-value < 0.05 was considered statistically significant.

### 2.8 Ethics statement

This study was reviewed and approved by the Research Ethics Committee of the Balearic Islands (Comité de Ética de la Investigación de las Illes Balears), under project code CEI: IB 5359/23 PI. Ethical approval was granted on 31 January 2024. All radiographs were anonymized prior to analysis, and the study was conducted in accordance with institutional guidelines and the Declaration of Helsinki.

## 3 Results

### 3.1 Demographic characteristics

A total of 527 panoramic radiographs were included in the final analysis. The sample was balanced by sex, with 250 men (47.4%) and 277 women (52.6%). The age range was 6–65 years, with a mean age of 35.0 ± 16.2 years and a median age of 33 years (IQR 21–49), allowing comparative evaluation across demographic categories (Table 1).

**Table 1:** Demographic characteristics of the study sample.

Variable	Total sample (*n* = 527)
Age, mean ± SD (years)	35.0 ± 16.2
Age, median (IQR)	33 (21–49)
Men, *n* (%)	250 (47.4)
Women, *n* (%)	277 (52.6)

Values are presented as mean ± standard deviation (SD), median (interquartile range, IQR), or absolute frequency with percentages.

### 3.2 Dental developmental anomalies

#### 3.2.1 Overall prevalence and distribution

At least one dental developmental anomaly was identified in 38.2% of patients (Table 2). Among anomaly-positive individuals, anomalies were more frequently confined to the mandible (41.3%, *n* = 83), followed by involvement of both jaws (33.8%, *n* = 68) and the maxilla only (24.9%, *n* = 50) as shown in Table 2. Mandibular-only anomalies were significantly more frequent than maxillary-only anomalies (41.3% vs. 24.9%, *p* < 0.05).

**Table 2:** Overall prevalence and jaw distribution of dental anomalies.

Finding	*n*/*N*	Prevalence % (95% CI)
Patients with ≥1 anomaly	201/527	38.2 (34.1–42.4)
One anomaly	146/527	27.7 (24.1–31.7)
Two anomalies	49/527	9.3 (7.1–12.1)
Three anomalies	6/527	1.1 (0.5–2.5)
Jaw distribution among anomaly-positive patients (*n* = 201)		
Location	*n*/*N*	% (95% CI)
Mandible only	83/201	41.3 (34.7–48.2)
Maxilla only	50/201	24.9 (19.4–31.3)
Both jaws	68/201	33.8 (27.3–40.3)

Prevalence values are reported with 95% confidence intervals (Wilson method). Jaw distribution percentages are calculated among anomaly-positive patients (*n* = 201).

No significant sex differences were observed: 37.6% of men (94/250) and 38.6% of women (107/277) presented at least one anomaly (*p* = 0.78), with odds ratio analysis confirming the absence of association (OR = 0.95; 95% CI: 0.67–1.35). Patients with anomalies were significantly younger, with a median age of 24 years. The highest prevalence was observed in the 15–25-year age group (Fisher's exact test, *p* < 0.001), and odds ratio analysis confirmed that individuals in this age group had significantly higher odds of presenting anomalies compared with the remaining age groups (OR = 4.68; 95% CI: 3.14–6.98).

Regarding multiplicity, most affected individuals presented a single anomaly: 27.7% (*n* = 146) had one anomaly, 9.3% (*n* = 49) had two, and 1.1% (*n* = 6) had three anomalies simultaneously. Consequently, the anomaly categories presented below are not mutually exclusive.

#### 3.2.2 Prevalence by anomaly category

When anomalies were grouped by type, eruption disturbances were the most frequent category. Because a patient could present more than one anomaly type, these categories were not mutually exclusive. Overall, eruption disturbances affected 32.3% of the sample, followed by shape anomalies (8.7%) and number anomalies (1.7%), as shown in Table 3.

**Table 3:** Prevalence of dental anomaly categories and main eruption disturbances.

Category	*n*/*N*	Prevalence % (95% CI)
Number anomalies	9/527	1.7 (0.9–3.2)
Shape anomalies	46/527	8.7 (6.6–11.4)
Eruption disturbances	170/527	32.3 (28.4–36.4)
Main eruption disturbance subtypes		
Subtype	*n*/*N*	Prevalence % (95% CI)
Impaction	142/527	26.9 (23.3–30.9)
Retention	66/527	12.5 (10.0–15.6)

Patient level prevalence values are presented as percentage of the total sample (*n* = 527) with 95% confidence intervals (Wilson method). Categories are not mutually exclusive because a patient could present more than one anomaly type.

##### 3.2.2.1 Anomalies of number

A total of 9 patients (1.7%) presented anomalies of number, including agenesis in 6 patients (1.1%) and supernumerary teeth in 3 patients (0.6%). Both findings were more frequent in women, although the differences were not statistically significant (*p* = 0.64). Most cases occurred in younger age groups; however, inferential analysis was limited due to the low number of cases.

**Figure 1:**
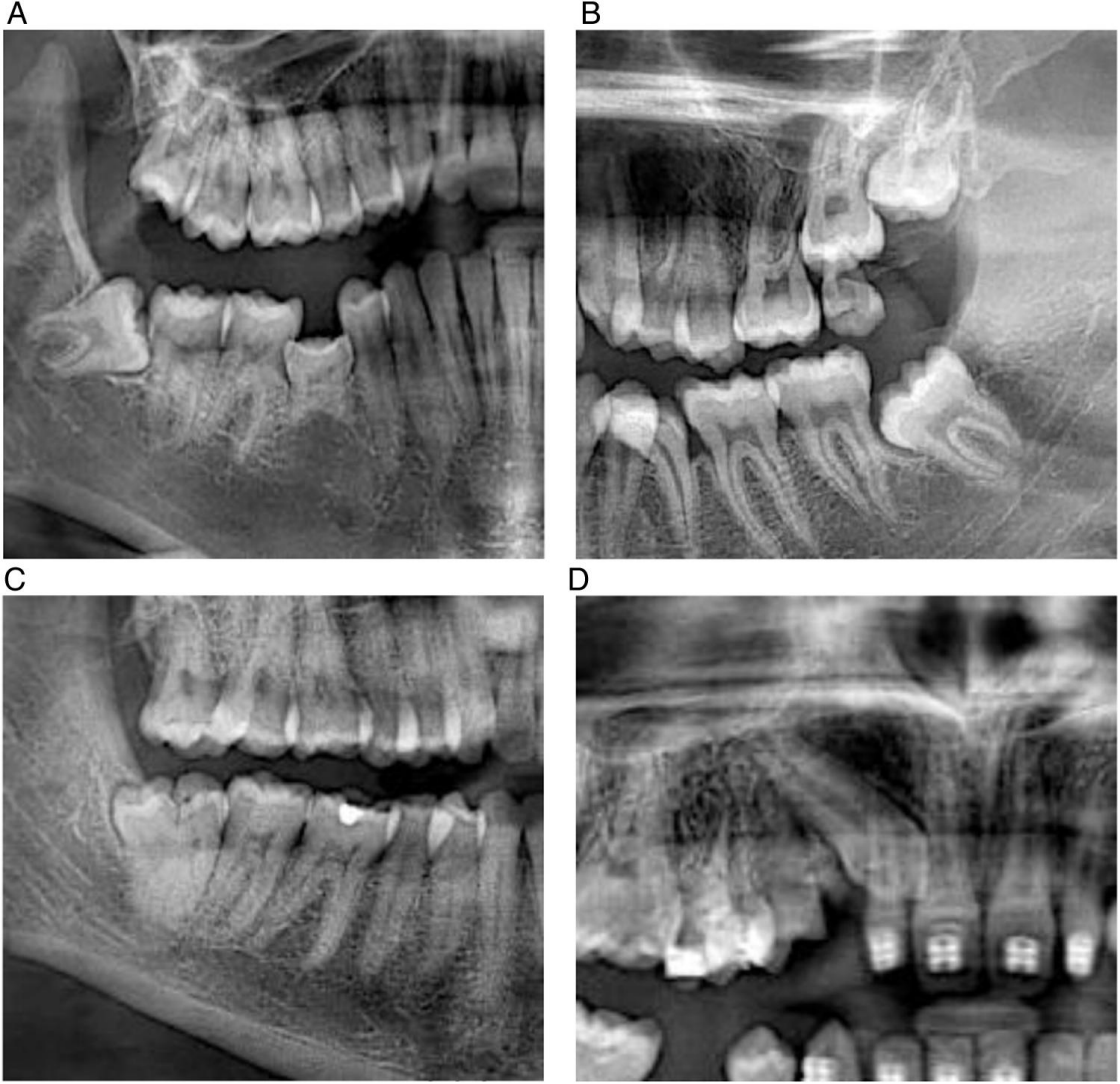
Representative panoramic radiographs of dental developmental anomalies. **(A)** Panoramic radiograph illustrating agenesis of the mandibular right second premolar (tooth 4.5) with an associated impacted mandibular right third molar (tooth 4.8). **(B)** Panoramic radiograph showing impacted maxillary and mandibular left third molars (teeth 2.8 and 3.8), as well as a supernumerary tooth preventing the eruption of tooth 2.7. **(C)** Panoramic radiograph demonstrating an impacted mandibular right third molar (tooth 4.8) with gemination, classified as a shape anomaly. **(D)** Panoramic radiograph illustrating an impacted maxillary right canine (tooth 1.3), classified as an eruption disturbance.

At the tooth level, 11 teeth were affected by number anomalies. Agenesis accounted for eight missing teeth, most frequently involving tooth 4.5 (*n* = 4) as shown in Figure 1A, followed by tooth 3.5 (*n* = 3) and tooth 1.5 (*n* = 1). Three supernumerary teeth were identified: one mandibular incisor and two in molar regions (Figure 1B), one mandibular and one maxillary.

##### 3.2.2.2 Anomalies of shape

Among shape anomalies, 46 patients (8.7%) presented at least one alteration. Dilaceration was the most frequent finding (*n* = 32; 69.6%), followed by taurodontism (*n* = 7; 15.2%), conoid teeth (*n* = 6; 13.0%), and gemination (*n* = 1; 2.2%). No cases of fusion or dens invaginatus were observed. Dilaceration was significantly more frequent in women (62.5% vs. 37.5%; *p* = 0.048).

At the tooth level, 58 teeth presented shape anomalies. Conoid teeth were observed in six teeth, most frequently affecting the maxillary lateral incisor (tooth 1.2; *n* = 3), followed by tooth 2.8 (*n* = 2) and tooth 2.2 (*n* = 1). Dilaceration affected 38 teeth and was mainly located in posterior teeth, including mandibular third molars (teeth 4.8 and 3.8) and the maxillary second premolar (tooth 2.5). Taurodontism was identified in 13 teeth, predominantly in mandibular second molars (teeth 4.7 and 3.7). A single case of gemination was detected in tooth 4.8, as shown in Figure 1C.

##### 3.2.2.3 Eruption disturbances

Eruption disturbances constituted the most prevalent anomaly category. Impactions were observed in 142 patients (26.9%), followed by retentions in 66 patients (12.5%) and ankylosis in one patient (0.2%). These categories were not mutually exclusive, and some patients presented more than one eruption disturbance. No significant sex differences were detected (*p* > 0.05). A strong age association was observed, with impactions significantly more frequent in individuals aged 15–30 years compared with other age groups (*p* < 0.001); retentions showed a similar distribution pattern (*p* < 0.001).

At the tooth level, 357 teeth presented eruption disturbances. Impacted teeth accounted for 261 cases and were predominantly mandibular third molars (Figures 1A–D), particularly teeth 3.8 and 4.8, followed by maxillary third molars (teeth 2.8 and 1.8). Less frequently, impactions were observed in canines (Figure 1D) and premolars, including teeth 2.3, 1.3, and 4.3. Retained teeth accounted for 95 cases and were mainly located in maxillary third molars (teeth 2.8 and 1.8), followed by mandibular third molars (teeth 3.8 and 4.8). A single case of ankylosis was identified in tooth 8.5.

### 3.3 Jawbone lesions

#### 3.3.1 Overall prevalence, location, and multiplicity

Jawbone lesions were identified in 30.0% of patients. Among lesion-positive individuals, 54 patients (10.2%) presented multiple lesions.

Lesions were predominantly located in the mandible. Among patients with lesions, 58.9% presented mandibular lesions only, 24.7% maxillary lesions only, and 16.5% lesions affecting both jaws, as detailed in Table 4. Mandibular lesions were significantly more frequent than maxillary lesions (58.9% vs. 24.7%, *p* < 0.05).

**Table 4:** Overall prevalence, multiplicity, and jaw distribution of jawbone lesions.

Finding	*n*/*N*	Prevalence % (95% CI)
Patients with ≥1 jaw lesion	158/527	30.0 (26.2–34.0)
Single lesion	104/527	19.7 (16.6–23.3)
Multiple lesions	54/527	10.2 (7.9–13.1)
Location among lesion-positive patients (*n* = 158)		
Location	*n*/*N*	% (95% CI)
Mandible only	93/158	58.9 (51.1–66.2)
Maxilla only	39/158	24.7 (18.6–32.0)
Both jaws	26/158	16.5 (11.5–23.2)

Prevalence values are presented with 95% confidence intervals (Wilson method). Location percentages are calculated among lesion-positive patients (*n* = 158).

No statistically significant sex differences were observed in lesion prevalence (32.5% in women vs. 27.2% in men; *p* = 0.186), with odds ratio analysis confirming the absence of association (OR = 0.78; 95% CI: 0.53–1.15) or lesion location by sex (*p* = 0.193). However, men showed a higher proportion of multiple lesions compared with women (42.6% vs. 27.8%), approaching statistical significance (*p* = 0.051).

Patients presenting lesions were significantly older than those without lesions (41.4 ± 15.1 vs. 32.3 ± 15.9 years; *p* < 0.001). Lesion prevalence increased significantly with age, rising from 14.7% in the 6–20-year group to 44.8% in patients aged 50–65 years (*p* < 0.001), with patients aged ≥35 years showing higher odds of lesions (OR = 3.28; 95% CI: 2.18–4.92).

#### 3.3.2 Radiodensity patterns

Considering the total number of lesions detected, radiolucent lesions predominated (Table 5). Radiolucent lesions accounted for 73.8% of all lesions, followed by radiopaque lesions in 24.5%, while mixed-density lesions were rare (1.7%).

**Table 5:** Radiographic characteristics of jawbone lesions and periapical radiolucent inflammatory patterns.

Radiodensity	*n*/*N*	% (95% CI)
Radiolucent	175/237	73.8 (67.9–79.0)
Radiopaque	58/237	24.5 (19.4–30.3)
Mixed	4/237	1.7 (0.7–4.3)
Periapical inflammatory lesions (lesion-positive patients, *n* = 158)		
Finding	*n*/*N*	% (95% CI)
Patients with radiolucent periapical inflammatory lesions	125/158	79.1 (72.1–84.7)

Radiodensity percentages are calculated based on the total number of lesions detected (*n* = 237). Periapical radiolucent inflammatory lesion prevalence is calculated among lesion-positive patients (*n* = 158). All confidence intervals were computed using the Wilson method.

Radiolucent lesions were significantly more frequent in the maxilla compared with the mandible (*p* < 0.001) and were more common in men, although this difference was not statistically significant (*p* = 0.089). By contrast, radiopaque lesions showed an opposite distribution, being significantly more frequent in the mandible (*p* < 0.001) and significantly more prevalent in women (*p* = 0.020). Patients with radiolucent lesions were significantly older than those with radiopaque lesions (*p* = 0.011).

#### 3.3.3 Location relative to teeth and prevalence of lesion types by radiographic criteria

Most lesions were in the periapical region. Among lesion-positive patients (*n* = 158), 125 individuals (79.1%) presented radiolucent inflammatory periapical lesions, which were significantly more frequent in men than in women (*p* = 0.04).

**Figure 2:**
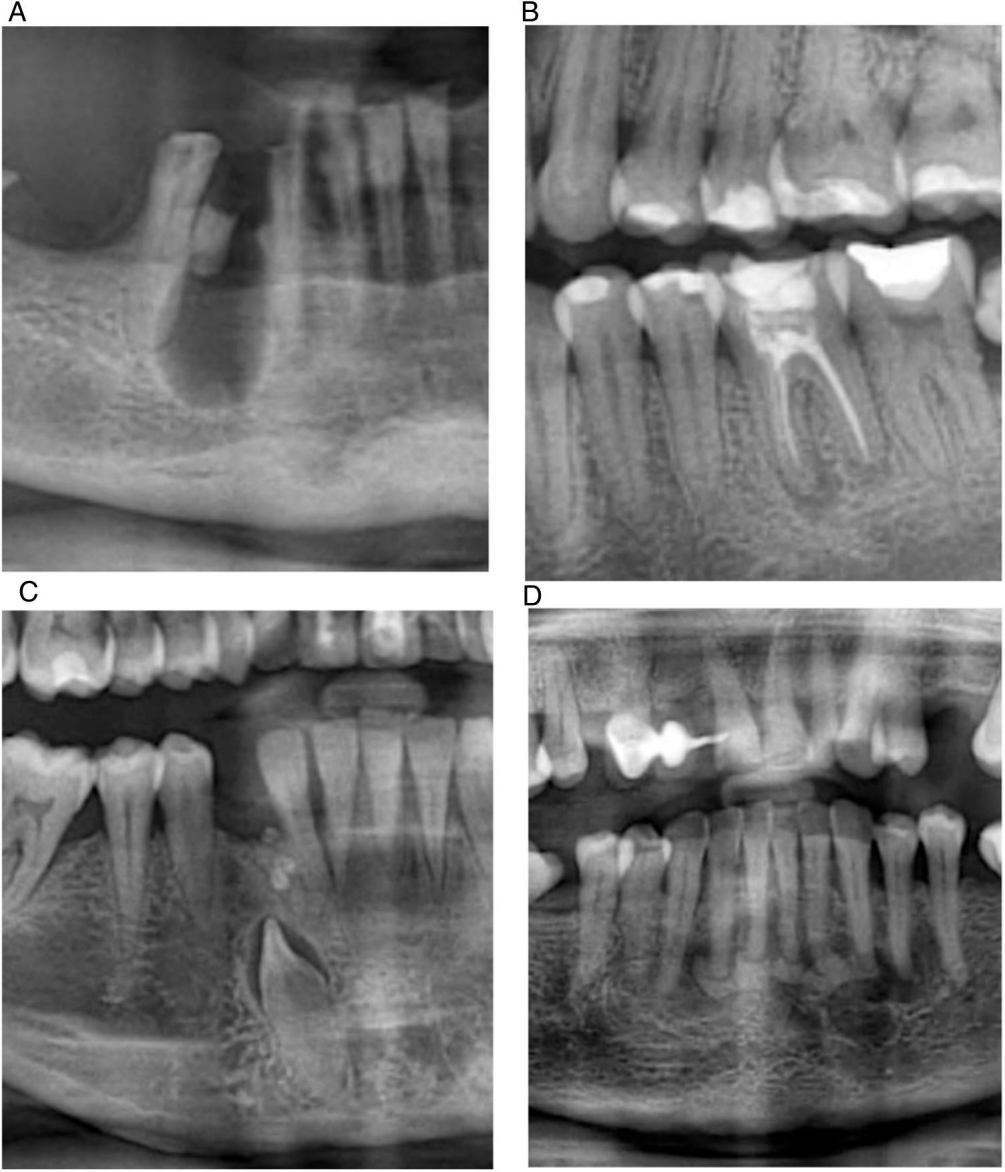
Representative panoramic radiographs of jawbone lesions. **(A)** Panoramic radiograph illustrating an apical radiolucent lesion consistent with an inflammatory radicular cyst associated with tooth 4.4. **(B)** Panoramic radiograph demonstrating a radiolucent lesion consistent with a periapical granuloma associated with tooth 3.6. **(C)** Panoramic radiograph illustrating a radiopaque lesion consistent with an odontoma and an associated impacted mandibular right canine (tooth 4.3). **(D)** Panoramic radiograph showing a periapical mixed-density lesion in the anterior mandibular region (teeth 3.5–4.3), suggestive of cemento-osseous dysplasia.

Based on radiographic criteria, 26 patients (16.5%) presented lesions consistent with radicular cysts (Figure 2A), characterized by well-defined, round/oval periapical radiolucencies with corticated borders, predominantly located in the mandible (63.9%). Additionally, 94 patients (59.5%) showed features compatible with periapical granulomas (Figure 2B), also more frequently observed in the mandible (60.3%). These radiographic categories should be regarded as presumptive and not definitive, as histopathological confirmation was not available.

Other less frequent findings included condensing osteitis in 18 patients (11.4%), odontomas in 2 patients (1.3%) as shown in Figure 2C, and cemento-osseous dysplasia in 1 patient (0.6%), identified based on suggestive panoramic radiographic patterns (Figure 2D). Categories were not mutually exclusive because some patients presented more than one lesion type.

Finally, first permanent molars were the most frequently involved teeth in periapical inflammatory lesions (*p* < 0.001).

## 4 Discussion

### 4.1 Principal findings

This study provides an integrated radiographic epidemiological overview of dental developmental anomalies and jaw lesions in a university dental clinic population from Mallorca.

The prevalence of dental developmental anomalies (38.2%) and jaw lesions (30.0%) falls within the ranges reported in panoramic radiographic studies, which vary according to population characteristics, diagnostic criteria and imaging protocols (2, 18–20, 22, 23).

Eruption disturbances, particularly third molar impactions, were the most frequent anomaly category, while inflammatory periapical lesions represented the predominant type of jaw pathology.

### 4.2 Anatomical and age distribution of dental anomalies

Beyond overall prevalence, dental anomalies showed a clear anatomical pattern. Among anomaly-positive patients, mandibular involvement predominated, with mandibular-only anomalies being significantly more frequent than maxillary-only anomalies (41.3% vs. 24.9%, *p* < 0.05). This mandibular predominance is likely related to the high frequency of eruption disturbances affecting mandibular third molars, which are commonly associated with altered eruption paths and space limitations (13, 14).

A clear age-related pattern was also observed. Patients presenting anomalies were significantly younger, with the highest prevalence concentrated in the 15–25-year age group. This distribution reflects the period during which eruption disturbances and orthodontic assessments are most frequently detected. Similar age-related patterns have been reported in panoramic studies evaluating dental anomalies, where most eruption disturbances are diagnosed during adolescence and early adulthood (13, 19, 21).

From a clinical perspective, these findings support the importance of systematic radiographic screening during adolescence and early adulthood, when eruption disturbances are most prevalent. Early identification may guide the timing of radiographic examinations, as well as orthodontic evaluation and surgical referral when indicated.

### 4.3 Eruption disturbances as the predominant anomaly category

When anomalies were analyzed by category, eruption disturbances represented the most prevalent anomaly group. Impactions and retentions were particularly frequent and showed significant associations with younger age groups. Since some individuals presented more than one eruption disturbance, subtype frequencies were not mutually exclusive.

The predominance of eruption disturbances, particularly third molar impactions, is consistent with previous epidemiological studies based on panoramic radiographs, which consistently identify impacted third molars as the most common developmental anomaly detected in dental practice (13, 14, 19). The high prevalence of impactions observed in the present study likely reflects both biological eruption patterns and clinical referral patterns in university dental clinics, where orthodontic and surgical consultations are common.

Early radiographic identification of eruption disturbances is relevant because impacted teeth may predispose to pericoronitis, odontogenic infection, cyst formation, resorption of adjacent roots, and periodontal complications (13, 32). Therefore, systematic radiographic assessment during adolescence may facilitate preventive management and timely surgical intervention when indicated.

### 4.4 Number and shape anomalies

Anomalies of number were relatively uncommon in the present cohort. The prevalence of agenesis was lower than that reported in some epidemiological studies, which may be explained by differences in diagnostic criteria, particularly regarding the inclusion or exclusion of third molars. Nevertheless, the distribution observed in this study, with premolars among the most frequently missing teeth, is consistent with previously reported patterns of hypodontia (3).

Supernumerary teeth were also rare in this cohort. This finding aligns with previous reports indicating that supernumerary teeth occur in a small proportion of the population and are frequently located in the anterior maxilla or molar regions (4).

Among anomalies of shape, dilaceration was the most frequently detected alteration and was significantly more prevalent in women. Dilaceration prevalence varies widely across studies due to differences in imaging modalities and diagnostic thresholds, with cone-beam computed tomography often revealing higher detection rates compared with panoramic radiography (6). Taurodontism was observed mainly in mandibular molars and at a prevalence within the lower range reported in the literature (7, 8). The absence of fusion and dens invaginatus likely reflects both their low epidemiological frequency and the limited sensitivity of panoramic imaging for subtle morphological alterations (9–12).

### 4.5 Prevalence and patterns of jawbone lesions

The bone lesion component complements the anomaly findings by characterizing jaw lesions within the same cohort. Jawbone lesions were identified in nearly one-third of patients (30%), closely matching prospective evidence reporting prevalence around 29% in panoramic radiographic studies (16). Lesions were significantly more frequent in older individuals, with prevalence increasing progressively across age groups and reaching its highest level in patients aged 50–65 years. This pattern is consistent with cumulative exposure to caries, restorative history, endodontic treatment, and other age-related risk factors associated with periapical pathology (27, 28).

Anatomically, mandibular lesions predominated, with mandibular involvement significantly more frequent than maxillary lesions (58.9% vs. 24.7%, *p* < 0.05). Radiolucent lesions accounted for most findings, which is consistent with epidemiological studies showing that inflammatory odontogenic lesions constitute the majority of radiographic jaw findings detected on panoramic imaging (22, 23).

Conversely, radiopaque lesions represented a smaller but clinically relevant proportion of findings and were significantly more frequent in the mandible and in female patients. Similar distributions have been reported in retrospective panoramic studies evaluating radiopaque jaw lesions, where mandibular predominance is commonly observed (29). Radiopaque lesions encompass a heterogeneous group of entities, including inflammatory conditions such as condensing osteitis as well as fibro-osseous lesions and odontogenic tumors. Accurate interpretation therefore requires careful assessment of radiographic characteristics such as location, borders, and internal structure (30, 33).

From a diagnostic perspective, differentiating radiopaque or mixed-density jaw lesions based solely on panoramic radiography remains challenging, and radiographic interpretation should always be integrated with clinical findings and, when necessary, additional imaging or histopathological evaluation (29, 30).

### 4.6 Inflammatory periapical lesions and clinical implications

Most lesions identified in the present study corresponded to radiolucent inflammatory periapical lesions, affecting 79.1% of lesion-positive patients. Radiographic patterns compatible with periapical granulomas and radicular cysts accounted for the majority of these lesions, although differentiation between these entities remains presumptive without histopathological confirmation (24–28).

The predominance of periapical inflammatory lesions observed in this study is consistent with previous epidemiological surveys of jaw lesions, where inflammatory odontogenic conditions constitute the majority of radiographically detected pathology (22, 23). The first permanent molars were the most frequently affected teeth involved in periapical lesions. This finding likely reflects their early eruption, prolonged functional exposure, and higher susceptibility to caries and pulpal infection.

Lesion prevalence increased with age, supporting the need for continued radiographic monitoring in adult populations, particularly in individuals with extensive restorative or endodontic history. These findings reinforce the value of age-adapted imaging protocols and systematic radiographic interpretation in optimizing preventive and clinical decision-making.

From a clinical standpoint, early detection of periapical pathology is essential, as it may influence endodontic treatment planning, tooth preservation strategies, and long-term oral health outcomes.

Beyond local effects, inflammatory periapical lesions may also have broader systemic implications. Emerging evidence suggests that apical periodontitis contributes to systemic inflammatory burden and may be associated with conditions such as cardiovascular disease, diabetes, and other chronic inflammatory disorders. Although systemic associations were not evaluated in this study, the high prevalence observed highlights the importance of early detection and management within a comprehensive approach to patient care (34, 35).

Within this context, it is also relevant to consider the potential interaction between jawbone lesions and dental developmental conditions. Although the coexistence of both entities was not a primary analytical objective of this study, their concurrent presence within the same population highlights the relevance of an integrated radiographic approach. In our sample, jawbone lesions were occasionally observed in association with altered eruption patterns, as illustrated in Figure 2C; however, these findings were infrequent and do not support a consistent association.

### 4.7 Limitations and strengths

Several limitations should be considered when interpreting the findings of this study. The retrospective design and convenience sampling may introduce selection bias, and panoramic radiography has inherent limitations in diagnostic specificity compared with cone-beam computed tomography or histopathological confirmation.

Additionally, tooth- and lesion-level analyses did not account for potential within-patient clustering, since multiple teeth or lesions could occur in the same individual. Individual risk factor data such as smoking, systemic health conditions, or socioeconomic variables were also unavailable, limiting etiological interpretation.

Despite these limitations, the study presents several strengths. Radiographs were obtained using a standardized imaging protocol and evaluated by calibrated examiners under the supervision of an experienced reference examiner. The sample included a broad age range and balanced sex distribution, and the simultaneous evaluation of dental developmental anomalies and jawbone lesions within the same cohort provides a comprehensive overview of radiographic findings in a university-clinic population.

### 4.8 Future directions

Overall, these findings support the diagnostic value of systematic panoramic evaluation and standardized reporting protocols in university-clinic settings, not only for epidemiological characterization but also for guiding preventive and clinical decision-making. Future multicenter studies incorporating broader demographic diversity, risk-factor profiling, and confirmatory diagnostic approaches where clinically indicated are warranted to refine prevalence estimates and improve etiologic understanding.

## 5 Conclusion

In this university dental clinic population from Mallorca, panoramic radiography revealed a substantial prevalence of both dental developmental anomalies and jawbone lesions, highlighting the clinical relevance of systematic radiographic assessment in routine practice.

Dental developmental anomalies were identified in more than one-third of patients, with eruption disturbances representing the most frequent category and occurring predominantly in younger individuals, particularly during late adolescence and early adulthood. In contrast, jawbone lesions were more commonly observed in older patients and were predominantly inflammatory and periapical in origin. These findings indicate age-related differences, with anomalies more frequent in earlier stages of dental development and lesions increasing with cumulative dental pathology.

The high frequency of incidental findings detected in this study underscores the importance of careful and systematic interpretation of panoramic radiographs in routine dental practice. Standardized radiographic evaluation protocols may support earlier detection of anomalies and jaw pathology, thereby improving clinical management.

Future multicenter studies including broader populations, detailed clinical risk-factor profiling, and confirmatory diagnostic methods such as cone-beam computed tomography or histopathological analysis when indicated are warranted to further refine prevalence estimates and improve the epidemiological understanding of radiographically detected dental anomalies and jawbone lesions.

## Data Availability

The datasets presented in this article are not readily available due to ethical and privacy restrictions but may be made available by the corresponding author upon reasonable request. Requests to access the datasets should be directed to Daniela Vallejos, d.vallejos@eua.edu.es.
